# A Community-Based Validation Study of the Short-Form 36 Version 2 Philippines (Tagalog) in Two Cities in the Philippines

**DOI:** 10.1371/journal.pone.0083794

**Published:** 2013-12-26

**Authors:** Nina T. Castillo-Carandang, Olivia T. Sison, Mary Lenore Grefal, Rody G. Sy, Oliver C. Alix, Elmer Jasper B. Llanes, Paul Ferdinand M. Reganit, Allan Wilbert G. Gumatay, Felix Eduardo R. Punzalan, Felicidad V. Velandria, E. Shyong Tai, Hwee-Lin Wee

**Affiliations:** 1 Department of Clinical Epidemiology, College of Medicine, and Institute of Clinical Epidemiology, National Institutes of Health, University of the Philippines (U.P.) Manila, Manila, Philippines; 2 LIFEcourse study in CARdiovascular disease Epidemiology (LIFECARE) Philippines Study Group, Lipid Research Unit, U.P.-Philippine General Hospital, U.P. Manila, Manila, Philippines; 3 Cardinal Santos Medical Center, San Juan City, Metro Manila, Philippines; 4 Department of Medicine, Yong Loo Lin School of Medicine, National University Health System, Singapore, Singapore; 5 Department of Pharmacy, National University of Singapore and Department of Rheumatology & Immunology, Singapore General Hospital, Singapore, Singapore; University of Tolima, Colombia

## Abstract

**Objective:**

To evaluate the validity and reliability of the Philippines (Tagalog) Short Form 36 Health Survey version 2 (SF-36v2^®^) standard questionnaire among Filipinos residing in two cities.

**Study Design and Setting:**

The official Philippines (Tagalog) SF-36v2 standard (4-week recall) version was pretested on 30 participants followed by formal and informal cognitive debriefing. To obtain the feedback on translation by bilingual respondents, each SF-36v2 question was stated first in English followed by Tagalog. No revisions to the original questionnaire were needed except that participants thought it was appropriate to incorporate "*po*" in the instructions to make it more polite. Face-to-face interviews of 562 participants aged 20-50 years living in two *barangays* (villages) in the highly urbanized city of Makati City (Metro Manila) and in urban and rural barangays in Tanauan City (province of Batangas) were subsequently conducted. Content validity, item level validity, reliability and factor structure of the SF-36v2 (Tagalog) were examined.

**Results:**

Content validity of the SF-36v2 was assessed to be adequate for assessing health status among Filipinos. Item means of Philippines (Tagalog) SF-36v2 were similar with comparable scales in the US English, Singapore (English and Chinese) and Thai SF-36 version 1. Item-scale correlation exceeded 0.4 for all items except the bathing item in PF (correlation: 0.31). In exploratory factor analysis, the US two-component model was supported. However, in confirmatory factor analysis, the Japanese three-component model fit the Tagalog data better than the US two-component model.

**Conclusions:**

The Philippines (Tagalog) SF-36v2 is a valid and reliable instrument for measuring health status among residents of Makati City (Metro Manila) and Tanauan City (Province of Batangas).

## Introduction

The Short Form 36 (SF-36) Health Survey is a generic instrument which assesses “functional health and well-being from the patient’s perspective”.[1] It is a 36-item questionnaire which has been translated to over 140 languages and is used globally to assess changes in health status as well as comparing the burden of illness in a population. The eight areas of perceived health in SF-36 include: Physical Functioning (PF), Role Physical (RP), Bodily Pain (BP), General Health (GH), Vitality (VT), Social Functioning (SF), Role Emotional (RE) and Mental Health (MH). The scores range from zero (0) to one hundred (100) with higher score representing better health status. 

Previous clinical trials and research studies in the Philippines have used version one of the SF-36[2-4]. However, there is an improved version known as the SF-36 version 2 (SF-36v2) where the instructions and questionnaires items were revised to improve their clarity. In SF-36v2, the following revisions were made: (1) simpler instructions and improved layout for questions and answers to improve clarity, (2) improved phrasing of some items to provide greater comparability with translations and cultural adaptations widely-used in the U.S. and in other countries and (3) revision of response options from dichotomous to five-level response choices for the role physical and role emotional items to improve sensitivity and from six-level to five-level response choices for the vitality and mental health items for simplification[5]. As any revisions to the SF-36v1 may be expected to alter its psychometric properties, we seek to perform a socio-cultural validation of the Philippines (Tagalog) SF-36v2 among urban and rural adults living in two cities in the Philippines. 

## Methods

### Study Design and Study Participants

 This was a cross-sectional community survey of adults aged 20-50 years old. The study was done in 3 phases: pre-testing, informal and formal cognitive debriefing/interviews and a community survey. Ethics approvals were obtained from the Cardinal Santos Medical Center and from the University of the Philippines Manila. Pretesting of SF-36 was conducted among thirty (30) participants of varied backgrounds (i.e. tricycle drivers, factory workers, students, housewife, midwife and clinical researchers) in purposively chosen communities (one rural and two urban communities). The main purpose of pre-testing was to identify difficult words and phrases. Informal cognitive interviews (n= 24, all self-administered Tagalog questionnaire) were conducted during which participants were asked about their overall feedback, feelings and perceptions about the Philippines (Tagalog) SF-36v2. Formal cognitive interviews (n=36, all interviewer-administered Tagalog and US (English) questionnaire) during which respondents assessed each individual item in the questionnaire were then conducted. During formal cognitive debriefing the following were asked for each question and its associated response options: (1) Did you have difficulty understanding this question/response choice? (2) What does this question/response choice mean to you? (3) Is the question/response choice relevant to your condition? (4) How would you have worded this question/response option? (5) Is the response option consistent with the question? The following were asked for the instructions on how to answer the questionnaire: (1) Did you have difficulty understanding this instruction? (2) What does this instruction mean to you? (3) How would you have worded this instruction? 

The community survey was conducted in 2 cities: (1) Makati which is the central business district of the Philippines, located 10 kilometers from the capital city of Manila; and (2) Tanauan (province of Batangas) which is 70 kilometers south of Manila. The selection of specific barangays was based on purposive sampling. Barangays were chosen with the assistance of the City Health Officers, and Planning and Development Officers of the local government units. The presence of local collaborators, accessibility, safety and security were primary considerations for choice of barangays. To the extent possible – some attempt was also made to reflect the diversity of barangays (e.g., rural and urban, major sources of household income, geographic location – lowland, coastal, upland, etc.). Makati is a 1^st^ class city (based on average annual income of 400 million pesos or more, approx. USD 9.1 million) with 33 barangays (113,418 households, average household size 4.5 adults or children)[6]. Tanauan, on the other hand, is a 2^nd^ class city (average annual income between 320 to 400 million pesos, approx. USD 7.3 million to USD 9.1 million) with 47 barangays (31,268- households, average household size of 5 adults or children). Two *barangays* or villages (with an estimated 11,000 households) in Makati and 8 barangays (2 in the city center and 6 in outlying areas; with approximately 10,000 households) in the city of Tanauan[7] were included in the study. Residents of Makati as well as those from the more highly urbanized barangays of Tanauan (as in other urban areas in the Philippines) mainly earned their income from employment in offices and factories and from business. However, as is typical in the Philippines, many rural residents of Tanauan derived their income from agriculture. Both cities were accessible, relatively safe and secure; and the local government unit (LGU) and local collaborators agreed to participate in the study. The participants were fluent in Filipino (Tagalog) or English and gave written informed consent. As part of a longitudinal study to evaluate the risk of developing cardiovascular diseases in the Philippines and three other Southeast Asian countries (Indonesia, Malaysia, Thailand)[8], participants who had existing cardiovascular disease as determined by participant’s medical history (previous myocardial infarction, stroke, peripheral arterial disease); had a history of malignancies (treated or otherwise); and had plans to migrate outside their community within the next 5 years were excluded. 

**Figure 1:**
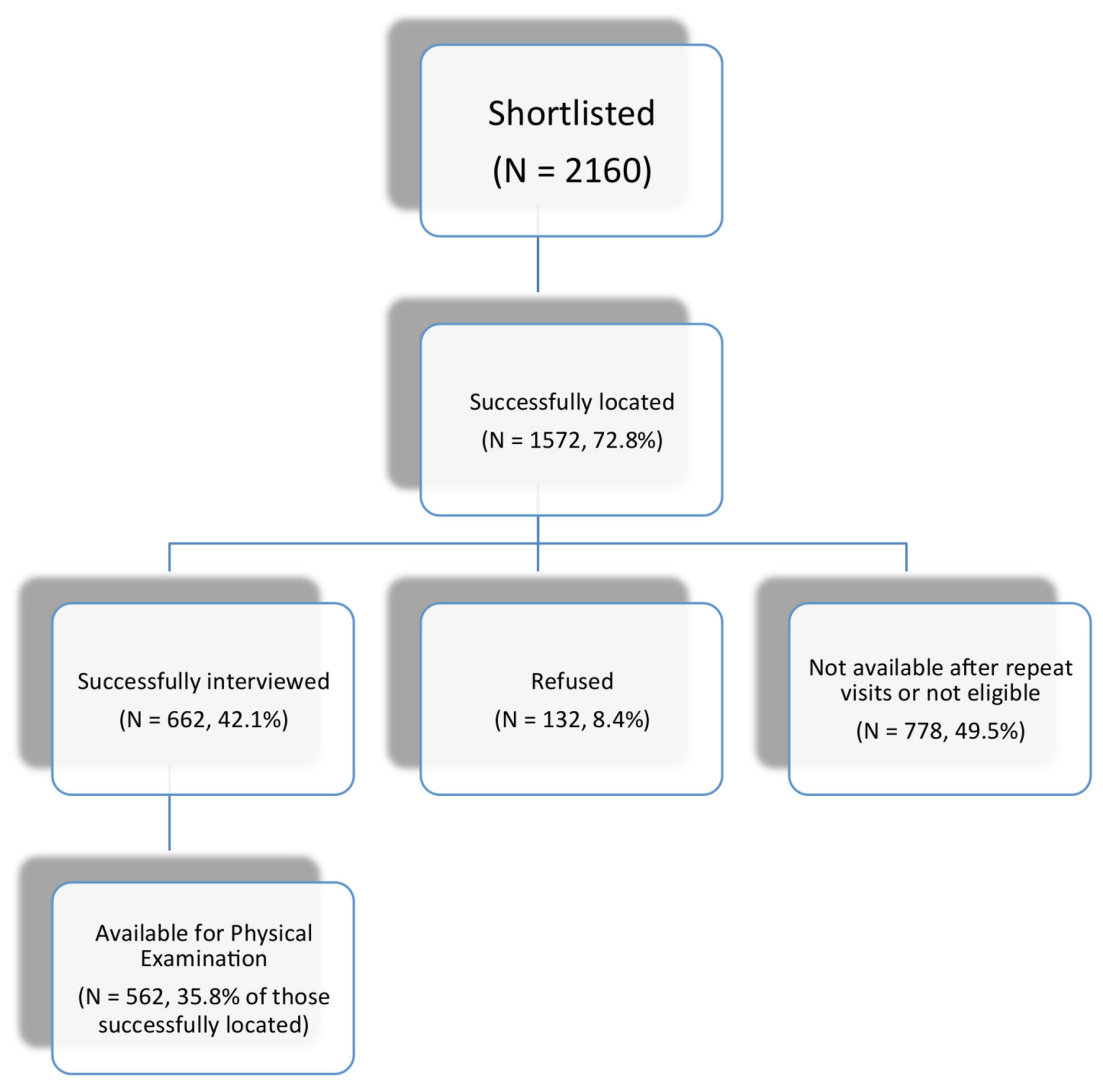
Flowchart on patient recruitment for the community survey.

The names of household heads were obtained from the LGUs and entered into Microsoft Excel 2007^®^ and duplicates were removed. Random sampling was performed using a random number table. A total of 2,160 households were selected with 300 households for each of the 2 *barangays* in Makati City and 120 households for each of the 8 barangays in Tanauan City. As it was more difficult to locate potential study participants in the highly urbanized barangays of Makati, we pre-selected a larger number of households in Makati. A list of household members was generated and the Kish method was used to randomly select only one person per household[9]. Five hundred sixty-two persons (180 in Makati and 382 in Tanauan; total response rate: 35.8% among those successfully contacted, Figure 1) agreed to participate in the study. 

 The team with the assistance of the *barangay* health worker or any knowledgeable local resident then located the selected households to ensure that the household member was indeed living in that household and that inclusion criteria were met. If the said household member was not present during the initial visit, an appointment was made for a face-to-face interview at their convenience. A maximum of 3 visits including weeknights and weekends was done before the selected member was considered unavailable. Written informed consent was usually obtained during the initial visit while most interviews were conducted during the third visit. 

### The Questionnaire

 In the questionnaire, each question and its corresponding response options were first presented in English and followed immediately by its Filipino (Tagalog) translation. Participants could choose between the English or Tagalog versions of the questionnaire. During the formal cognitive interview which is interviewer-administered, once the participant had decided on the language version, the interviewer would read the questionnaire in one language only. For bilingual respondents, they were asked to evaluate both English and Tagalog versions. Nonetheless, all of them chose to complete the questionnaire in Tagalog. We are aware that this is not the typical presentation of different language versions of SF-36v2 questionnaires. However, this was considered a pragmatic compromise to reduce the number of survey questionnaires that an interviewer needs to carry around. Unlike in other major cities, transportation may be challenging in certain parts of Makati and Tanauan, particularly during monsoon season when floods are common. Although the SF-36v2 is designed for self-administration, prior experience in other research projects informed us that face-to-face interviews with the use of cue cards is more appropriate due to the limitations posed by the literacy level of some respondents. 

### Data Confidentiality

When data was collected, each participant was identified using only a code. The files linking the code to the subject identifiers were kept in a separate file from the data. Only the Principal Investigator and the data manager have access to this linking file. All data were kept on a computer that is password protected. All other study investigators (both within and without the Philippines) were allowed access to de-identified data only, and only after written permission from the Principal Investigator was obtained. 

#### Content Validity

Three aspects of validity were assessed and these were content validity, item level validity and construct validity. Item level validity and construct validity are described in the statistical analyses section. Content validity, also known as “content relevance” or “content coverage,” evaluates whether the questions (content domain) in a measurement tool are appropriate relative to its intended use. Clarity, comprehensiveness and redundancy of items and scales of an instrument are evaluated. There are usually no standards against which it can be measured statistically as it is based more on previous research and on lay and expert opinion. 

The ultimate aim of the scale is that we can infer from the final scores and draw valid conclusions about the population that had been studied. The higher the content validity of a measure, the broader the inferences we can draw. The questions that make up the SF-36v1 have been validated in many languages and socio-cultural contexts and are deemed suitable for assessing health status. Content validity was assessed through eliciting expert opinions of the Philippine LIFECARE research team (5 cardiologists, 1 health social scientist, 1 nutritionist, 1 medical technologist) as well as feedback from lay persons during the informal and formal cognitive interviews.

### Statistical Analyses

#### Ceiling and floor effects

The percentage of respondents achieving maximum and minimum scores were examined as this has impact on the sensitivity and responsiveness of an instrument. For example, if a respondent has achieved the maximum possible score of 100 (ceiling), then any improvement in his/her health status cannot be picked up by the instrument. Similarly, if a respondent has achieved the lowest possible score of 0 (floor), then further deterioration of his/her health status cannot be picked up by the instrument. When the floor and ceiling effects are high, the instrument has limited value for measuring changes in health status or discriminating between respondents with small differences in health status. Based on published Singapore SF-36v1 data[10], it was hypothesized that ceiling effects would be 30-40% for PF, BP and SF and less than 5% for GH, VT and MH. It was also hypothesized that with the increase in response options from 2 to 5 on RP and RE, the observed ceiling effects of approximately 70% in the Singapore SF-36v1 data will be reduced to 30-40%, in line with other scales using the 5-level response option. Similarly, based on published Singapore English SF-36v1 data[10], it was hypothesized that floor effects would be less than 1% on all scales. In the Singapore English SF-36v1, floor effects for RP and RE were approximately 10%. The authors believed that this will reduce to approximately 1% with the revised number of response options. 

 Item level validity of the SF-36v2 would be supported if Likert scale scoring assumptions were fulfilled: (1) Item means and standard deviations being similar within each scale, (2) Item – scale correlations > 0.4 and of similar magnitude within each scale and (3) Successful tests of item discriminant validity (correlation between an item and its hypothesized scale being higher than the correlations between that item and other scales). Item means and standard deviation of the Philippines (Tagalog) SF-36v2 were compared with published data on four scales (PF, BP, GH and SF) from the Singapore (English)[10], Thailand[11], Japan[12] and US (English) SF-36v1[13]. The remaining four scales of SF-36v1 (RP, RE, MH and VT) were excluded from the comparison because the number of response choices increased from 2 to 5 in RP and RE while the number of response choices decreased from 6 to 5 in MH and VT in SF-36v2. 

#### Construct validity

 Both exploratory (EFA) and confirmatory factor analyses (CFA) were performed to evaluate the factor structure of SF-36v2 (Tagalog). As the names suggest, EFA is generally employed when the factor structure is unknown whereas CFA is used to confirm an *a priori* factor structure[14]. In EFA, principal component analysis with varimax rotation was performed and it was specified that two factors be extracted. This is in accordance with the US two-component model. In CFA, two separate models were evaluated. The first CFA model was based on the published US two-component model for the SF-36v2[5] while the second CFA was based on the published Japanese three-component model [15]. Given that the sociocultural context of the Philippines is likely to be more similar to Japan than the US, we hypothesized that the three-component model would fit the Philippines data better than the US two-component model. The following goodness-of-fit statistics were used to compare the two CFA models: Akaike information criterion (AIC), Bayesian information criterion (BIC), likelihood ratio (LR), root mean square error of approximation (RMSEA), adjusted Satorra-Bentler variance estimates, comparative fit index (CFI) and Tucker-Lewis index (TLI). With AIC, BIC, LR, RMSEA and adjusted Satorra-Bentler variance, smaller values indicate better model fit. With CFI and TLI, values closer to 0.95 or more indicate better model fit[16].

#### Reliability

 This study measured reliability by measuring internal consistency or homogeneity (i.e., the degree to which a group of items in a domain or scale measure the same characteristic). High internal consistency connotes greater reliability of the score. It is deemed optimal for the items to be moderately correlated with each other and that each item should correlate with the total scale score. An instrument is considered reliable for measurements at the group level if Cronbach’s alpha exceeded 0.7 and reliable for measurements at the individual level if Cronbach’s alpha exceeded 0.9. 

## Results

### Pre-testing, Cognitive Debriefing/Interviews and Content Validity

 The key take home message from pre-testing was that the SF-36v2, which was designed to be self-administered, should be interviewer-administered. There were no changes to the contents of SF-36v2. The experts and lay persons who participated in either the pre-testing or cognitive interviews all agreed that the items in the Philippines (Tagalog) SF-36v2 were mostly understandable and were relevant to a general assessment of a Filipino’s health status. In the informal Cognitive Debriefing (n=24), 42% were males and 58% were females. Most of the respondents were blue collar workers. In the formal Cognitive Debriefing (n=36), 28% were males and 72% were females. Mean (SD) age was 34 years. More than half (56%) were unemployed, 39% had some college education (but were not college graduates) and 36% were high school graduates. Sixteen of the 36 respondents (44%) felt that the questionnaire was too long. 

Most of the participants in both formal and informal cognitive interviews said they were comfortable answering the SF-36v2 and gave no suggestions for re-wording them. Bilingual respondents found the translation adequate. Interestingly, many respondents expressed difficulty with the PF items. The authors’ general experience with the SF-36v2 in other countries is that respondents would have problems with RE and MH items. Nonetheless in general the Philippines respondents found the SF-36v2 items easy to understand. Respondents found it easier if the tool was interviewer-administered vs. self-administered as is the usual method of administration of SF-36v2. The questions and the responses were deemed to be easy to understand and there were no changes made except for the need to make the introduction to the questionnaire and the initial instructions sound “polite” by adding the word “*po*” so as to be culturally apt in the course of the face-to-face interview. Average time for the total interview (including demographic questions and time taken to clarify if respondents understood the questions) was 36 minutes. 

### Community Survey for Validation of SF-36v2^®^

 Five hundred sixty-two respondents participated in the cross-sectional community survey (Table 1). Majority (62%) were females; mean age for both sexes was 36 years; 64% were married; and 41% had at least college education. Thirty-eight percent were unemployed, 35% employed, 24% self-employed and the rest were retirees and students. 

**Table 1:** Demographic characteristics of 562 respondents.

		**Number (%), unless otherwise stated**		
		**Makati City**	**Tanauan**	**Total**
**Sex**				
	Male	53 (29.4)	160 (41.9)	213 (37.9)
	Female	127 (70.6)	222 (58.1)	349 (62.1)
**Mean (SD**)** Age**		35.3 (9.0)	35.7 (8.8)	35.6 (8.9)
	Median (IQR)	36.0 (14)	36.0 (16)	36.0 (15)
**Civil Status**				
	Single	50 (27.8)	93 (24.4)	143 (25.4)
	Married	107 (59.4)	255 (66.8)	362 (64.4)
	Widow/widower	3 (1.7)	3 (0.8)	6 (1.1)
	Separated	2 (1.1)	3 (0.8)	5 (0.9)
	Live-in	18 (10.0)	28 (7.3)	46 (8.2)
**Highest Educational Attainment**				
	Some Elementary	2 (1.1)	12 (3.1)	14 (2.5)
	Elementary Graduate	4 (2.2)	29 (7.6)	33 (5.9)
	Some High School	7 (3.9)	33 (8.6)	40 (7.1)
	High School Graduate	52 (28.9)	140 (36.7)	192 (34.2)
	Vocational Course Graduate	9 (5.0)	41 (10.7)	50 (8.9)
	Some College	62 (34.4)	54 (14.1)	116 (20.6)
	College Graduate	44 (24.4)	72 (18.8)	116 (20.6)
	Postgraduate	0	1 (0.3)	1 (0.2)
**Employment Status**				
	Employed	47 (26.1)	149 (39.0)	196 (34.9)
	Self-employed	24 (13.3)	113 (29.6)	137 (24.4)
	Retired	2 (1.1)	1 (0.3)	3 (0.5)
	Student	8 (4.4)	5 (1.3)	13 (2.3)
	Unemployed	99 (55.0)	114 (29.8)	213 (37.9)

#### Ceiling and Floor Effects

As hypothesized, ceiling effects were moderate for SF (31.5%), RP (32.7%) and RE (39.2%). The remaining scales had minimal ceiling effects ranging from 2 to 19 percent, which was better than hypothesized. On the other hand, floor effects were absent in all scales except for one respondent each in SF and RE (Table 2).

**Table 2:** Distribution of the Philippines (Tagalog) SF-36v2 scores.

**SF-36v2 scales**	**Philippines (Tagalog**)** SF-36v2**						
	**Mean**	**SD**	**Median (range)**	**Skewness**	**Floor/Ceiling (%)**	**Item-scale correlation**	**Item discriminant correlation range**
**PF**	83.07	16.64	85.00 (5-100)	-1.4	0.00/19.04	0.31-0.71	0.08-0.38
**RP**	78.84	20.67	81.25 (12.5 - 100)	-0.66	0.00/32.74	0.79-0.83	0.21-0.56
**BP**	68.09	20.82	62 (22 - 100)	0.21	0.00/21.71	0.85-0.93	0.28-0.44
**GH**	70.55	17.64	72 (10 - 100)	-0.45	0.00/1.96	0.57-0.75	0.08-0.36
**VT**	71.59	15.33	71.88 (18.75 - 100)	-0.18	0.00/5.52	0.59-0.69	0.17-0.47
**SF**	77.80	19.17	75 (0 - 100)	-0.35	0.18/31.49	0.81-0.87	0.24-0.43
**RE**	79.86	20.72	83.33 (0 - 100)	-0.67	0.18/39.15	0.85-0.89	0.17-0.57
**MH**	80.11	14.82	80 (15 - 100)	-0.66	0.00/13.52	0.58-0.67	0.12-0.48

#### Item level validity

 Item means and standard deviations were similar within each scale in the Philippines (Tagalog) SF-36v2 (Table S1). In addition, item-scale correlation exceeded 0.4 for all except one PF item (bathing) where item-scale correlation was 0.31 (Table S2). Furthermore, item-other scale correlations were poor, thus providing evidence for item discriminant validity. For example, item-other scale correlations between PF items and other scales ranged from 0.08 to 0.38 (Table 2). Hence, item level validity of the Philippines (Tagalog) SF-36v2 was supported. 

#### Construct validity

 At the scale level, mean scores ranged from 68.09 (BP) to 83.07 (PF) (Table 2). Philippine norm-based scores were not computed as this sample is not representative of the Philippines Tagalog-speaking general population. However, if the US norms[5] were applied, then this study sample has scores that are very similar to the US general population (Table 3). 

**Table 3:** Philippines (Tagalog) SF-36v2 Norm-based Scores.

**Scale**	**Philippines (Tagalog) Mean (0-100 scale)**	**US Mean (0-100 scale)**	**US Standard Deviation (0-100 scale)**	**Philippines (Tagalog) Norm-based scores based on the US general population**
PF	85	84.2	23.3	50.03
RP	81.25	80.9	34	50.01
BP	62	75.2	23.7	49.44
GH	72	71.9	20.3	50.00
VT	71.88	60.9	20.9	50.53
SF	75	83.3	22.7	49.63
RE	83.33	81.3	33	50.06
MH	80	74.7	18.1	50.29

Ref. Ware JE. SF-36® Health Survey Update. Retrieved from http://www.sf-36.org/tools/SF36.shtml/

 Item level factor analysis of the 36 items extracted eight factors, which explained 57 percent of the total variance (Table S3). The first factor explained 25 percent of the total variance, while the other factors were less significant, each explaining only less than 10 percent of the total variance. In the Singapore (English) SF-36v1, item level factor analysis yielded seven factors instead of eight. 

 In EFA, the Philippines (Tagalog) factor structure is very similar to the Singapore (English) and Japan SF-36v1 and rather distinct from the US (English) SF-36v1 (Table 4). This provided further support that the Japanese 3-component model may be a better fit to this dataset than the US two-component model. Indeed, in CFA, the Japanese 3-component model was superior with goodness of fit index being 0.933 and 0.833 for the Japanese and US models, respectively (Table 5). With regards to reliability, the SF-36v2 Filipino version exhibited good internal consistency with Cronbach’s alpha coefficient exceeding the recommended value of 0.70 for all scales except GH, VT and SF (Table 6). 

**Table 4:** Scale level factor analysis of the Philippines (Tagalog) SF-36v2.

**SF-36v2 scales**	**Hypothesized association**		**Correlation with rotated principal components**											
			**Philippines (Tagalog**)** SF-36v2**			**Singapore (English) SF-36v1**			**Japan SF-36v1**			**United States (English**)** SF-36v1** ^†^		
	Physical	Mental	Physical	Mental	*h^2^*	Physical	Mental	*h^2^*	Physical	Mental	*h^2^*	Physical	Mental	*h^2^*
**PF**	●	O	**0.66**	0.20	0.48	**0.60**	0.14	0.38	**0.61**	0.25	0.56	**0.85**	0.12	0.74
**RP**	●	O	**0.86**	0.17	0.76	**0.85**	0.12	0.74	**0.94**	0.22	0.67	**0.81**	0.27	0.73
**BP**	●	O	**0.48**	**0.46**	0.44	**0.46**	**0.53**	0.49	**0.44**	**0.47**	0.54	**0.76**	0.28	0.66
**GH**	◑	◑	0.10	**0.75**	0.57	0.14	**0.74**	0.57	0.36	**0.60**	0.54	**0.69**	0.37	0.61
**VT**	◑	◑	0.24	**0.81**	0.71	0.15	**0.84**	0.73	0.26	**0.82**	0.71	**0.47**	**0.64**	0.63
**SF**	◑	●	**0.56**	0.34	0.43	**0.49**	**0.56**	0.55	**0.43**	**0.50**	0.54	**0.42**	**0.67**	0.63
**RE**	O	●	**0.78**	0.22	0.66	**0.77**	0.18	0.73	**0.69**	0.40	0.51	0.17	**0.78**	0.64
**MH**	O	●	0.28	**0.74**	0.62	0.12	**0.83**	0.70	0.22	**0.82**	0.75	0.17	**0.87**	0.79

Principal components extraction with varimax rotation. Factor loadings ≥ 0.40 were considered significant and are in bold.

*h* ^*2*^ = proportion of total variance of each scale explained by the two extracted components

●: Strong association (r ≥ 0.70)

◑: Moderate to substantial association (0.30 < r < 0.70)

O: Weak association (r ≤ 0.30)

^†^ presented US SF-36v1 data as the authors did not have access to US SF-36v2 data.

**Table 5:** Confirmatory factor analyses for the Philippines (Tagalog) SF-36v2.

Fit indices	Model 1: Based on Published United States Factor Structure	Model 2: Based on Hypothesized Japanese Factor Structure
AIC	0.00038	0.00037
BIC	0.00038	0.00038
LR	216.170	103.011
RMSEA (90% CI)	0.1360 (0.1200, 0.1526)	0.0950 (0.0778, 0.1130)
SBadj	172.247	85.338
CFI	0.8326	0.9334
TLI	0.7533	0.8903

Abbreviations – AIC: Akaike information criteria; BIC: Schwarz Bayesian information criteria; CFI: comparative fit index; CI: confidence interval; LR: Log likelihood ratio; RMSEA: root mean squared error of approximation; SBadj: Adjusted Satorra-Bentler variance estimates; TLI: Tucker-Lewis index

**Table 6:** Spearman’s correlation and internal consistency reliability of the Philippines (Tagalog) SF-36v2 scores.

	**SF-36v2 scale**							
	**PF**	**RP**	**BP**	**GH**	**VT**	**SF**	**RE**	**MH**
**Philippines (Tagalog)**								
**PF**	**(0.86)**							
**RP**	0.48	**(0.82)**						
**BP**	0.40	0.44	**(0.78)**					
**GH**	0.33	0.31	0.34	**(0.62)**				
**VT**	0.36	0.36	0.41	0.43	**(0.51)**			
**SF**	0.30	0.44	0.37	0.33	0.35	**(0.54)**		
**RE**	0.34	0.62	0.36	0.22	0.41	0.44	**(0.83)**	
**MH**	0.31	0.35	0.34	0.35	0.59	0.35	0.44	**(0.87)**

## Discussion

 To the best of the authors’ knowledge, this is the first paper to report the reliability and validity of the Philippines (Tagalog) SF-36v2. Ceiling effect was within expected level while floor effect was almost non-existent. Content, item level and construct validity were supported. For example, item means and standard deviations of Philippines (Tagalog) SF-36v2 for PF, BP, GH and SF were highly similar to the item means and standard deviations of corresponding SF-36v1 scales from three other countries. Similar to the Singapore (English) SF-36v1, VT and MH items overlapped on two factors instead of all VT items loading onto one factor and all MH items loading onto another factor. Unlike the Singapore English SF-36v1, the Philippines (Tagalog) SF-36v2 PF items loaded onto two factors rather than one, thus generating one more factor compared to the Singapore (English) SF-36v1. In addition, it was confirmed that the Japanese three-component model better described the Philippines data compared to the US two-component model, lending further support to the need for a different model in Asia. Although the internal consistency of three Philippines (Tagalog) SF-36v2 scales were below threshold, it was noted that similar observations were made with the Singapore (English) SF-36v1[10] where Cronbach’s alpha were 0.67 and 0.58 for VT and SF, respectively. In the US English SF-36v2[5], internal consistency of SF was borderline at 0.68. This is probably because the SF scale comprises only two items. Due to insufficiently high Cronbach’s alpha, the authors cautioned on the use of GH, VT and SF scales independently but supported their use as part of Physical Component Summary Score (PCS) and Mental Component Summary Score (MCS). PCS and MCS are two summary scales derived from the eight scales of SF-36v2. 

 This paper is important in several ways. First, the psychometric properties of the Philippines (Tagalog) SF-36v2 have not been previously evaluated. Given that 96% of people living in the Philippines can speak Tagalog[17], this study has provided important information for a questionnaire that is likely to be used widely in the Philippines. Second, the data in this study were compared with those from the region (Singapore, Thailand and Japan) and found that the Philippines (Tagalog) SF-36v2 performed similarly with these countries. This is important for multinational clinical trials in Asia as it implies that meaningful cross-country comparisons may be made since the same concept of health status is being measured across various countries. 

 A potential limitation of this study is that the sample is not representative of the Tagalog-speaking general population in the Philippines. Our subjects were aged 20-50 years old. When compared to the respective population of the same age band, our sample reflects the population with regards to the highest education attained but had an over-representation of female participants and slight under-representation of married participants. However, based on the authors’ experience in conducting this study in two cities, it is likely that conducting such a population-based study will be a mammoth task requiring huge amount of resources, particularly as the Philippines is made up of more than 7,100 islands. The authors have tried to mitigate this by sampling from both rural and urban populations. In addition, the response rate may limit the authors’ ability to generalize the findings. There were two major reasons for the apparently low response rate. First, response rate was computed as a percentage of those who were successfully located. Many of those who were successfully located were at work even though the interviewers tried to visit on different times of the day or both weekdays and weekends. If the response rate was computed as a percentage of those who were successfully located and eligible, then it will be a high response rate of 70.8%. It should be pointed out that upfront refusal was only 8.4% among those successfully located. The second reason was that the eventual sample size was based on a combination of willingness to be interviewed as well as availability for physical examination. 100 of those who were willing to be interviewed were not available for physical examination due to their work schedule. However, the authors believe that the response rate is comparable to or even better than other similar door-to-door surveys. In the Global Study of Sexual Attitudes and Behaviors which includes the Philippines as a study site, the mean overall response rate was 19%, and ranged from 8–55% in the various countries[18].

 In conclusion, the results of this study support the reliability and validity of the SF-36v2 Philippines (Tagalog) for assessing health status among Tagalog-speaking urban and rural adults aged 20-50 years old in Makati and Tanauan. 

## Supporting Information

Table S1 **Item means and standard deviations (SD).**
(DOCX)

Table S2 **Spearman item-scale correlations of the Philippines (Tagalog) SF-36v2.**
(DOCX)

Table S3 **Item level factor analysis of the Philippines (Tagalog) SF-36v2.**
(DOCX)
