# Supplementary material for: A Community-Based Validation Study of the Short-Form 36 Version 2 Philippines (Tagalog) in Two Cities in the Philippines
Source: PLoS One. 2013 Dec 26;8(12):e83794. doi: 10.1371/journal.pone.0083794 (PMC3873385; doi:10.1371/journal.pone.0083794)
Supplement: Table S1 — Item means and standard deviations (SD). (DOCX) [file pone.0083794.s001.docx]

**Supplementary Table S1. Item means and standard deviations (SD)**

| **SF-36 items clustered in the hypothesized order** |  |  | **Mean (SD)** | | | | |
| --- | --- | --- | --- | --- | --- | --- | --- |
|  |  | **Philippines**  **Tagalog**  **V2** | | **Singapore English**  **V1** | **Japan**  **V1** ^†^ | **Thailand**  **V1** | **United States**  **English v1** |
| **Physical functioning (PF)** |  |  | |  |  |  |  |
| SF36_3 Vigorous activities | 3a | 2.28 (0.57) | | 2.26 (0.71) | 2.10 | 1.99 (0.64) | 2.17 |
| SF36_6 Climbing several flights | 3d | 2.51 (0.60) | | 2.46 (0.67) | 2.67 | 2.51 (0.63) | 2.54 |
| SF36_8 Bending, kneeling or stooping | 3f | 2.63 (0.53) | | 2.55 (0.67) | 2.79 | 2.56 (0.59) | 2.59 |
| SF36_9 Walking more than one kilometer | 3g | 2.56 (0.59) | | 2.54 (0.68) | 2.75 | 2.22 (0.73) | 2.55 |
| SF36_4 Moderate activities | 3b | 2.67 (0.52) | | 2.57 (0.63) | 2.80 | 2.58 (0.57) | 2.65 |
| SF36_5 Lifting/carrying groceries | 3c | 2.59 (0.55) | | 2.57 (0.66) | 2.80 | 2.56 (0.61) | 2.72 |
| SF36_10 Walking several hundred meters | 3h | 2.76 (0.47) | | 2.59 (0.65) | 2.90 | 2.65 (0.58) | 2.69 |
| SF36_7 Climbing one flight | 3e | 2.80 (0.45) | | 2.67 (0.61) | 2.89 | 2.74 (0.50) | 2.78 |
| SF36_11 Walking one hundred meters | 3i | 2.85 (0.39) | | 2.65 (0.64) | 2.94 | 2.76 (0.50) | 2.82 |
| SF36_12 Bathing or dressing | 3j | 2.95 (0.27) | | 2.70 (0.67) | 2.97 | 2.90 (0.33) | 2.88 |
| **Role physical (RP)*** |  |  | |  |  |  |  |
| SF36_14 Accomplished less | 4b | 4.14 (1.02) | | 1.78 (0.42) | 1.85 | 1.82 (0.38) | 1.73 |
| SF36_13 Cut down time on work | 4a | 4.13 (1.01) | | 1.80 (0.40) | 1.89 | 1.83 (0.38) | 1.83 |
| SF36_15 Limited in kind of work | 4c | 4.18 (1.02) | | 1.77 (0.42) | 1.79 | 1.88 (0.33) | 1.78 |
| SF36_16 Difficulty performing work | 4d | 4.16 (1.05) | | 1.81 (0.39) | 1.88 | 1.76 (0.43) | 1.77 |
| **Bodily pain (BP)** |  |  | |  |  |  |  |
| SF36_21 Intensity of bodily pain | 7 | 4.41 (1.16) | | 5.05 (1.14) |  | 4.51 (1.11) | 4.78 |
| SF36_22 Extent pain interfered with work | 8 | 4.40 (1.14) | | 4.69 (1.22) |  | 4.24 (0.76) | 4.58 |
| **General health (GH)** |  |  | |  |  |  |  |
| SF36_1 Your health is excellent…poor | 1 | 3.37 (0.84) | | 3.51 (0.84) | 2.99 | 3.07 (0.78) | 3.77 |
| SF36_34 As healthy as anybody | 11b | 3.44 (1.33) | | 3.80 (0.97) | 3.90 | 3.96 (0.98) | 3.80 |
| SF36_36 Health is excellent | 11d | 4.03 (1.11) | | 3.69 (0.94) | 3.61 | 3.58 (1.09) | 3.72 |
| SF36_33 Seem to get sick a little easier | 11a | 4.04 (1.18) | | 3.93 (1.04) | 3.72 | 3.76 (1.12) | 4.19 |
| SF36_35 Expect health to get worse | 11c | 4.22 (1.08) | | 3.94 (1.04) | 3.53 | 3.80 (1.12) | 3.66 |
| **Vitality (VT)*** |  |  | |  |  |  |  |
| SF36_23 Feel full of life | 9a | 4.02 (0.97) | | 4.35 (1.18) | 4.45 | 3.75 (0.93) | 3.82 |
| SF36_27 Have a lot of energy | 9e | 3.93 (1.03) | | 4.04 (1.24) | 3.94 | 3.62 (0.98) | 3.82 |
| SF36_29 Feel worn out | 9g | 4.18 (0.95) | | 4.49 (1.17) | 4.64 | 4.52 (0.88) | 4.34 |
| SF36_31 Feel tired | 9i | 3.32 (0.89) | | 4.07 (1.15) | 4.14 | 4.55 (0.91) | 4.02 |
| **Social functioning (SF)** |  |  | |  |  |  |  |
| SF36_32 Frequency social activities interfered | 10 | 4.01 (0.99) | | 4.02 (1.04) |  | 3.94 (0.98) | 4.25 |
| SF36_20 Extent social activities interfered | 6 | 4.22 (0.87) | | 4.33 (0.94) |  | 4.31 (0.75) | 4.35 |
| **Role emotional (RE)*** |  |  | |  |  |  |  |
| SF36_18 Accomplish less | 5b | 4.15 (0.95) | | 1.77 (0.42) | 1.84 | 1.77 (0.42) | 1.75 |
| SF36_17 Cut down amount of time on work | 5a | 4.19 (0.98) | | 1.79 (0.41) | 1.88 | 1.78 (0.42) | 1.84 |
| SF36_19 Did not do work as carefully | 5c | 4.24 (0.95) | | 1.81 (0.39) | 1.79 | 1.87 (0.34) | 1.82 |
| **Mental health (MH)*** |  |  | |  |  |  |  |
| SF36_26 Felt calm and peaceful | 9d | 3.98 (1.01) | | 4.19 (1.31) | 4.26 | 3.30 (0.93) | 4.06 |
| SF36_30 Been a happy person | 9h | 4.26 (0.86) | | 4.55 (1.17) | 3.96 | 4.11 (0.98) | 4.43 |
| SF36_24 Been very nervous | 9b | 4.07 (1.00) | | 4.82 (1.17) | 4.68 | 4.35 (0.85) | 4.85 |
| SF36_28 Felt so depressed | 9c | 4.25 (0.91) | | 4.95 (1.19) | 5.23 | 4.92 (0.92) | 5.33 |
| SF36_25 Felt downhearted and low | 9f | 4.45 (0.87) | | 4.74 (1.13) | 5.04 | 4.85 (0.92) | 4.98 |

Hypothesized item-cluster ordering follows that of the IQOLA project (Thumboo et al. 2001)

*Comparison of item scores on these scales across the language versions is not valid because in version 2, the number of response options for RP and RE were increased from 2 to 5 while that for MH and VT were reduced from 6 to 5.

† Only means for scales with three or more items are available. Reference. Gandek et al.Tests of data quality, scaling assumptions, and reliability of the SF-36 in eleven countries: results from the IQOLA Project. International Quality of Life Assessment. J Clin Epidemiol. 1998 Nov;51(11):1149-58.
