# Supplementary material for: A Community-Based Validation Study of the Short-Form 36 Version 2 Philippines (Tagalog) in Two Cities in the Philippines
Source: PLoS One. 2013 Dec 26;8(12):e83794. doi: 10.1371/journal.pone.0083794 (PMC3873385; doi:10.1371/journal.pone.0083794)
Supplement: Table S2 — Spearman item-scale correlations of the Philippines (Tagalog) SF-36v2. (DOCX) [file pone.0083794.s002.docx]

| **Supplementary Table S2. Spearman item-scale correlations of the Philippines (Tagalog) SF-36v2** | | | | | | | | | |
| --- | --- | --- | --- | --- | --- | --- | --- | --- | --- |
| Item | | Spearman item-scale correlations | | | | | | | |
| Name | Label | **PF** | **RP** | **BP** | **GH** | **VT** | **SF** | **RE** | **MH** |
| **PF (Physical Functioning)** | |  |  |  |  |  |  |  |  |
| 3a | Vigorous | **0.65** | 0.28 | 0.21 | 0.28 | 0.25 | 0.18 | 0.18 | 0.19 |
| 3b | Moderate | **0.65** | 0.38 | 0.27 | 0.16 | 0.24 | 0.28 | 0.30 | 0.17 |
| 3c | Lifting | **0.71** | 0.35 | 0.30 | 0.24 | 0.25 | 0.21 | 0.25 | 0.22 |
| 3d | Climb several flights | **0.70** | 0.35 | 0.28 | 0.22 | 0.24 | 0.21 | 0.25 | 0.23 |
| 3e | Climb one flight | **0.58** | 0.29 | 0.25 | 0.12 | 0.19 | 0.22 | 0.22 | 0.15 |
| 3f | Bending | **0.56** | 0.28 | 0.26 | 0.18 | 0.24 | 0.13 | 0.21 | 0.21 |
| 3g | Walk more than a kilometer | **0.71** | 0.34 | 0.27 | 0.25 | 0.29 | 0.23 | 0.24 | 0.24 |
| 3h | Walk several hundred meters | **0.62** | 0.31 | 0.21 | 0.21 | 0.18 | 0.17 | 0.23 | 0.20 |
| 3i | Walk one hundred meters | **0.52** | 0.32 | 0.19 | 0.13 | 0.15 | 0.19 | 0.24 | 0.17 |
| 3j | Bathing | **0.31** | 0.24 | 0.09 | 0.08 | 0.08 | 0.13 | 0.21 | 0.16 |
| **RP (Role Physical)** | |  |  |  |  |  |  |  |  |
| 4a | Cut down | 0.34 | **0.80** | 0.33 | 0.21 | 0.26 | 0.37 | 0.50 | 0.26 |
| 4b | Accomplished | 0.31 | **0.83** | 0.34 | 0.25 | 0.27 | 0.39 | 0.51 | 0.28 |
| 4c | Limited | 0.44 | **0.82** | 0.36 | 0.25 | 0.29 | 0.38 | 0.48 | 0.28 |
| 4d | Difficult | 0.45 | **0.79** | 0.44 | 0.27 | 0.36 | 0.35 | 0.56 | 0.33 |
| **BP (Bodily Pain)** | |  |  |  |  |  |  |  |  |
| 7 | Magnitude | 0.35 | 0.37 | **0.93** | 0.31 | 0.36 | 0.28 | 0.30 | 0.31 |
| 8 | Interfere | 0.37 | 0.44 | **0.85** | 0.29 | 0.38 | 0.39 | 0.37 | 0.30 |
| **GH (General Health)** | |  |  |  |  |  |  |  |  |
| 1 | General | 0.22 | 0.23 | 0.26 | **0.57** | 0.28 | 0.19 | 0.14 | 0.19 |
| 11a | Easier | 0.29 | 0.28 | 0.27 | **0.61** | 0.27 | 0.28 | 0.24 | 0.29 |
| 11b | Healthy | 0.15 | 0.14 | 0.13 | **0.66** | 0.25 | 0.19 | 0.08 | 0.13 |
| 11c | Worse | 0.16 | 0.15 | 0.22 | **0.61** | 0.27 | 0.17 | 0.10 | 0.25 |
| 11d | Excellent | 0.25 | 0.24 | 0.26 | **0.75** | 0.36 | 0.25 | 0.17 | 0.33 |
| **VT (Vitality)** | |  |  |  |  |  |  |  |  |
| 9a | Life | 0.17 | 0.19 | 0.21 | 0.28 | **0.64** | 0.25 | 0.22 | 0.37 |
| 9e | Energy | 0.25 | 0.19 | 0.25 | 0.32 | **0.69** | 0.18 | 0.24 | 0.44 |
| 9g | Worn | 0.29 | 0.32 | 0.34 | 0.26 | **0.61** | 0.26 | 0.37 | 0.47 |
| 9i | Tired | 0.23 | 0.24 | 0.25 | 0.24 | **0.59** | 0.24 | 0.24 | 0.29 |
| **SF (Social Functioning)** | |  |  |  |  |  |  |  |  |
| 6 | Extent | 0.28 | 0.42 | 0.34 | 0.28 | 0.28 | **0.81** | 0.43 | 0.31 |
| 10 | Time | 0.24 | 0.35 | 0.30 | 0.28 | 0.31 | **0.87** | 0.35 | 0.29 |
| **RE (Role Emotional)** | |  |  |  |  |  |  |  |  |
| 5a | Cut down | 0.29 | 0.55 | 0.29 | 0.17 | 0.35 | 0.38 | **0.87** | 0.40 |
| 5b | Accomplished | 0.31 | 0.57 | 0.36 | 0.21 | 0.35 | 0.37 | **0.89** | 0.35 |
| 5c | Not careful | 0.28 | 0.51 | 0.31 | 0.20 | 0.38 | 0.42 | **0.85** | 0.38 |
| **MH (Mental Health)** | |  |  |  |  |  |  |  |  |
| 9b | Nervous | 0.23 | 0.27 | 0.27 | 0.19 | 0.30 | 0.23 | 0.31 | **0.62** |
| 9c | No cheer | 0.30 | 0.35 | 0.24 | 0.22 | 0.35 | 0.31 | 0.43 | **0.66** |
| 9d | Calm | 0.21 | 0.22 | 0.20 | 0.27 | 0.44 | 0.27 | 0.23 | **0.67** |
| 9f | Down | 0.18 | 0.22 | 0.23 | 0.24 | 0.40 | 0.20 | 0.31 | **0.64** |
| 9h | Happy | 0.15 | 0.12 | 0.19 | 0.28 | 0.48 | 0.19 | 0.20 | **0.58** |
