# Supplementary material for: A Community-Based Validation Study of the Short-Form 36 Version 2 Philippines (Tagalog) in Two Cities in the Philippines
Source: PLoS One. 2013 Dec 26;8(12):e83794. doi: 10.1371/journal.pone.0083794 (PMC3873385; doi:10.1371/journal.pone.0083794)
Supplement: Table S3 — Item level factor analysis of the Philippines (Tagalog) SF-36v2. (DOCX) [file pone.0083794.s003.docx]

**Supplementary Table S3. Item level factor analysis of the Philippines (Tagalog) SF-36v2**

|  | **Factor loadings of individual SF-36 items after varimax rotation** | | | | | | |  |
| --- | --- | --- | --- | --- | --- | --- | --- | --- |
|  | **F1** | **F2** | **F3** | **F4** | **F5** | **F6** | **F7** | **F8** |
| **Physical functioning (PF)** |  |  |  |  |  |  |  |  |
| Vigorous activities (3a) |  |  |  |  |  | 0.76 |  |  |
| Moderate activities (3b) |  |  |  |  |  | 0.63 |  |  |
| Lifting/carrying groceries (3c) |  | 0.40 |  |  |  | 0.61 |  |  |
| Climbing several flights (3d) |  | 0.45 |  |  |  | 0.49 |  |  |
| Climbing one flight (3e) |  | 0.65 |  |  |  |  |  |  |
| Bending, kneeling or stooping (3f) |  | 0.57 |  |  |  |  |  |  |
| Walking more than one kilometer (3g) |  | 0.60 |  |  |  |  |  |  |
| Walking several hundred yards (3h) |  | 0.78 |  |  |  |  |  |  |
| Walking one hundred yards (3i) |  | 0.82 |  |  |  |  |  |  |
| Bathing or dressing (3j) |  | 0.66 |  |  |  |  |  |  |
| **Role physical (RP)** |  |  |  |  |  |  |  |  |
| Cut down time on work (4a) | 0.69 |  |  |  |  |  |  |  |
| Accomplished less (4b) | 0.73 |  |  |  |  |  |  |  |
| Limited in kind of work (4c) | 0.66 |  |  |  |  |  |  |  |
| Difficulty performing work (4d) | 0.65 |  |  |  |  |  |  |  |
| **Bodily pain (BP)** |  |  |  |  |  |  |  |  |
| Intensity of bodily pain (7) |  |  |  |  | 0.78 |  |  |  |
| Extent pain interfered with work (8) |  |  |  |  | 0.80 |  |  |  |
| **General health (GH)** |  |  |  |  |  |  |  |  |
| Your health is excellent…poor (1) |  |  |  |  |  |  | 0.58 |  |
| Seem to get sick a little easier (11a) |  |  |  |  |  |  | 0.49 |  |
| As healthy as anybody (11b) |  |  |  |  |  |  | 0.62 |  |
| Expect health to get worse (11c) |  |  |  |  |  |  | 0.46 |  |
| Health is excellent (11d) |  |  |  |  |  |  | 0.73 |  |
| **Vitality (VT)** |  |  |  |  |  |  |  |  |
| Feel full of life (9a) |  |  | 0.71 |  |  |  |  |  |
| Have a lot of energy (9e) |  |  | 0.70 |  |  |  |  |  |
| Feel worn out (9g) |  |  |  | 0.67 |  |  |  |  |
| Feel tired (9i) |  |  |  | 0.40 |  |  |  |  |
| **Social functioning (SF)** |  |  |  |  |  |  |  |  |
| Extent social activities interfered (6) |  |  |  |  |  |  |  | 0.53 |
| Frequency social activities interfered (10) |  |  |  |  |  |  |  | 0.68 |
| **Role emotional (RE)** |  |  |  |  |  |  |  |  |
| Cut down amount of time on work (5a) | 0.73 |  |  |  |  |  |  |  |
| Accomplish less (5b) | 0.75 |  |  |  |  |  |  |  |
| Did not do work as carefully (5c) | 0.69 |  |  |  |  |  |  |  |
| **Mental health (MH)** |  |  |  |  |  |  |  |  |
| Been very nervous (9b) |  |  |  | 0.52 |  |  |  |  |
| Felt so depressed (9c) |  |  |  | 0.63 |  |  |  |  |
| Felt calm and peaceful (9d) |  |  | 0.61 |  |  |  |  |  |
| Felt downhearted and low (9f) |  |  |  | 0.68 |  |  |  |  |
| Been a happy person (9h) |  |  | 0.74 |  |  |  |  |  |
| **Eigen value** | 8.61 | 2.85 | 2.20 | 1.47 | 1.40 | 1.16 | 1.10 | 0.98 |
| **% of Total Variance Explained** | 24.6 | 8.2 | 6.3 | 4.2 | 4.0 | 3.3 | 3.1 | 2.2 |
| **Cumulative % of Total Variance Explained** | 24.6 | 32.8 | 39.0 | 43.2 | 47.2 | 50.6 | 53.7 | 56.5 |
